# Knowledge, Attitude, and Practices of Indian Rheumatologists Regarding Reproductive Rheumatology in Female Patients: A Web-Based Survey

**DOI:** 10.31138/mjr.130724.arh

**Published:** 2025-06-30

**Authors:** Madhuri H Radhakrishna, Sunitha Kayidhi, Vinod Ravindran

**Affiliations:** 1 Star Hospital, Telangana, India;; 2 Continental Hospitals, Telangana, India;; 3 Department of Rheumatology, Centre for Rheumatology, Kozhikode, Kerala, India; 4 Department of Medicine, Kasturba Medical College, Manipal Academy of Higher Education, Manipal, Karnataka, India

**Keywords:** reproductive rheumatology, survey, knowledge, physician practices, India

## Abstract

**Background:**

Managing pregnancy and other reproductive concerns in individuals with rheumatic disease depends on the type of disease, its activity, co-morbidities, and other factors. This study’s primary objective was to evaluate rheumatologists’ knowledge, attitudes, and practices in this field.

**Methods:**

This cross-sectional web-based online survey was conducted from October to November 2022 among Indian Rheumatologists. Information was sought regarding knowledge and attitudes in managing pregnancy and reproductive health-related issues, their approach, barriers faced, patient perspectives, and possible solutions.

**Results:**

In total, 122 rheumatologists participated in the survey. Most rheumatologists were familiar with the British Society of Rheumatology (BSR) 2023 and American College of Rheumatology (ACR) 2020 guidelines on prescribing drugs in pregnancy and breastfeeding. Contraception and drug compatibility with breastfeeding were answered appropriately by 80% of the participants. Most (77.9%) were moderately to extremely confident in managing pregnant patients with rheumatic diseases. However, the majority were only slightly or not at all confident in discussing oocyte preservation(63%) and assisted reproductive techniques(65.9%). Almost all reported that their patients had fears about planning a pregnancy due to the disease (92%), and they frequently came across patients prematurely stopping their medicines after pregnancy was confirmed (50.8%).The majority of the respondents (91.7%) felt lack of proper treatment and regular follow-ups were the main contributing factors to poor pregnancy outcomes.

**Conclusion:**

Rheumatologists treating patients with reproductive issues in rheumatic diseases have certain knowledge gaps; filling these gaps can boost their confidence in managing these patients. There is scope for improving collaboration with obstetricians, and patient education.

## INTRODUCTION

Autoimmune rheumatic diseases (AIRDs) are frequently seen in women in the reproductive age of life. With better disease control, more women with AIRDs are contemplating and seeking information about pregnancy. They commonly approach their primary physicians, rheumatologists, and obstetricians with their queries and concerns. Pre-conception counselling, contraception advice, disease status review, and appropriate medications are integral to managing such pregnancies. A close collaboration between rheumatologists and obstetricians with relevant expertise is essential. Research in the field of reproductive rheumatology is fast expanding, and international guidelines for managing pregnancy, lactation, and other reproductive issues in AIRDs are being regularly updated.^1,2^ However, there are lacunae in the rheumatologists’ knowledge about the safety of drugs and choices for contraception.^3^ Despite feeling a sense of responsibility, rheumatologists are reluctant to advise their patients on family planning.^4^ In an Italian study on women with AIRDs, approximately one-third of the participants reported never receiving counselling regarding reproductive health from their doctors. These women expressed a preference for smaller families because they were worried about the impact of their disease on their unborn children and pregnancy. It was also noted that counselling had a favourable effect on family planning.^5^

Not only the knowledge and awareness regarding relevant aspects of reproductive rheumatology but also variable access to rheumatology services may negatively impact the outcomes. These aspects remain poorly explored in India which shares many relevant attributes with other countries of Southeast Asia.

This study’s primary objective was to evaluate Indian rheumatologists’ knowledge, awareness, attitudes, and practices in this field of reproductive rheumatology for female patients.

## METHODS

### Survey

This was a cross-sectional, web-based survey conducted among rheumatologists based in India between October 2022 and November 2022. A Google-form-based questionnaire was prepared and subjected to a face validity appraisal among a group of 10 doctors from other specialties who checked the usability and the technical functionality of the questionnaire, and the survey was accordingly modified. A recheck was carried out by the same group to confirm its technical functionality.

The survey with 40 questions was then subjected to pilot testing for content validity among 15 rheumatologists. Test-retest reliability for this pilot phase was tested using kappa statistics. The survey was anonymous, voluntary, and took about 15 minutes to complete. It was disseminated using e-mails and various social media platforms among rheumatologists. Participants who answered all questions were only included in the study. No incentives were provided to the participants.

### Questionnaire

The final version of the survey included 39 questions with 6 sections - Section 1 covered general information about the participants, section 2 knowledge of the participants, section 3 confidence and attitudes of participants, section 4 rheumatologists’ practices, section 5 patient beliefs and Section 6 about rheumatologists’ opinions (**Appendix**).

Basic demographics of the participating rheumatologists and commonly managed patient profiles were collected. They were asked to rate their familiarity with topics such as the ACR 2020 and BSR 2023 guidelines, the Pregnancy and Lactation Labelling Final Rule (PLLR) as defined by the US FDA definition, and specific questions about drug safety and methods of contraception. Their confidence level in dealing with questions about pregnancy management, contraception, assisted reproductive techniques (ART), and oocyte preservation methods was enquired into. Their practical experience in discussing conflicting advice given by other clinicians regarding safe drugs in pregnancy and difficulties in collaboration with other specialists was explored. Issues related to patient preferences and beliefs were also included in the questionnaire. Finally, suggestions for improving patient care were sought.

### Statistical analysis

Data entry was managed using Microsoft Excel 2019 version and analysis using EPI INFO version 7.2. Descriptive statistics were used to summarise data. For categorical variables frequencies and percentages were reported.

### Ethical approval

The Institutional Ethics Committee, AIG Hospital, Hyderabad gave the ethical approval (IEC-GH/2022/004).

## RESULTS

The responses to each domain are summarised as below.

### Demographics

A total of 122 responses were recorded, with the majority being males (89/122) in the age group of 31–40 years. Most were practicing in an urban setting (105/122) and were practising adult rheumatology (100/122). Access to full-time obstetrics services was sub-optimal (60%). Seventy-nine percent (96/122) of them were seeing at least 3 reproductive issue-related patients per month. Rheumatoid arthritis (RA) was the most common (75/122) rheumatological condition reported (**Table 1**).

**Table 1.** Demographic details of the participants.

**Characteristics**	**Answers**	**N (%)**

What is your age?	Between 31–40 years	65 (53%)
	Between 41–50 years	28 (23%)
	Between 51–60 years	19 (16%)
	More than 60 years	10 (8%)

What is your predominant area of practice?	Adult Rheumatologist	100 (82%)
	Paediatric Rheumatologist	03 (2.5%)
	Both adults and paediatric Rheumatologist	03 (2.5%)
	Physicians practicing Rheumatology	10 (8%)
	Trainees	03 (5%)

What is your predominant area of practice?	Urban	105 (86%)
	Rural	17 (14%)

What is your predominant practice setting?	Private clinic	44 (36%)
	Teaching institute	38 (31%)
	Corporate Hospital	37 (30%)
	Others (government / charitable hospital)	03 (3%)

How many years of practice do you have?	Less than 3 years	18 (15%)
	Between 3–7 years	35 (29%)
	More than 7 years	69 (57%)

Do you have a full-time Obstetrician available at your workplace?	Yes	73 (60%)
	No	49 (40%)

What is the average number of patients with reproductive issues seen by you per month?	Less than 3 per month	26 (21%)
	Between 3–7 per month	61 (50%)
	More than 7 per month	35(29%)

What is the most common rheumatic disease you encounter in pregnant patients?	Rheumatoid Arthritis	75 (62%)
	Systemic Lupus Erythematosus	34 (28%)
	Spondyloarthritis	05 (4%)
	Antiphospholipid Antibody Syndrome (APLA)	04 (3%)
	Others	04 (3%)

### Knowledge

Eighty-one participants (66.4%) were moderately and extremely familiar with the ACR 2020 guidelines, and a similar number (80/122) with the BSR 2023 guidelines on prescribing drugs in pregnancy and breastfeeding. A lesser number (53/122, 43%) reported similar familiarity with the PLLR on the prescription of drugs and biologics during pregnancy and breastfeeding. Appropriate contraception methods and drug compatibility with breastfeeding were answered accurately by 84% of the participants (**Table 2**).

**Table 2.** Responses to the questions in the knowledge domain.

**Characteristics**	**Answers**	**N (%)**

Are you familiar with ACR 2020 guidelines for the management of reproductive health in rheumatic and musculoskeletal diseases?	Extremely familiar	31 (25%)
	Moderately familiar	50 (41%)
	Slightly familiar	11 (9%)
	Somewhat familiar	22 (18%)
	Not at all familiar	08 (7%)

Are you familiar with the Pregnancy and Lactation Labelling Final Rule (PLLR) as defined by the US FDA for the prescription of drugs and biologics during pregnancy and breastfeeding?	Extremely familiar	19 (15%)
	Moderately familiar	34 (28%)
	Slightly familiar	17 (14%)
	Somewhat familiar	24 (20%)
	Not at all familiar	28 (23%)

Are you familiar with the BSR 2022 guidelines on prescribing drugs in pregnancy and breastfeeding?	Extremely familiar	34 (28%)
	Moderately familiar	46 (38%)
	Slightly familiar	14 (12%)
	Somewhat familiar	14 (12%)
	Not at all familiar	14 (12%)

Which USFDA category for the safety of drugs in pregnancy does Prednisolone come under?	Category A	15 (12%)
	Category B	45 (37%
	Category C	47 (39% )
	Category D	14 (12%)
	Category X	01 (1%)

Which of the following methods of contraception is preferred in APLA patients?	Progesterone IntraUterine Device	76 (63%)
	Depot Medroxy- Progesterone Acetate	25 (21%)
	Barrier method	06 (5%)
	Transdermal patch	05 (4%)
	Combined Estrogen and Progesterone Pill	06 (5%)
	Intrauterine contraceptive device	01 (1%)

Which of the following drugs is not compatible with breastfeeding?	Mycophenolate Mofetil	100 (82%)
	Azathioprine	09 (7%)
	Colchicine	07 (6%)
	Tacrolimus	06 (5%)

For which among the following patients do you routinely screen for anti-R0 and anti-La antibodies before planning pregnancy?	All patients with arthritis and connective tissue diseases	72 (59%)
	SLE	29 (24%)
	Sjogren's syndrome	19 (16%)
	SLE/Sjogren’s/myositis	1 (1%)
	I don’t routinely screen	1 (1%)

### Confidence and attitude

Confidence levels reported by the participants were relatively lower in discussing oocyte preservation techniques; 45/122 (37%) were not at all or slightly confident and ART (42/122, 35%). However, they reported better confidence in managing pregnancy, use of biologics, and discussing contraception methods (**Table 3**).

**Table 3.** Responses to the questions in the Confidence and attitude domain.

**Characteristics**	**Answers**	**N (%)**

How confident are you in managing pregnancy in rheumatological conditions?	Extremely confident	24 (20%)
	Moderately confident	71 (58%)
	Slightly confident	06 (5%)
	Somewhat confident	19 (16%)
	Not at all confident	02 (2%)

How confident are you in using Biologics during pregnancy?	Extremely confident	19 (16%)
	Moderately confident	43 (35%)
	Slightly confident	13 (11%)
	Somewhat confident	24 (20%)
	Not at all confident	23 (19%)

Are you confident in discussing contraception methods with your patients?	Extremely confident	36 (30%)
	Moderately confident	57 (47%)
	Slightly confident	08 (6%)
	Somewhat confident	19 (16%)
	Not at all confident	02 (2%)

Are you confident in discussing oocyte preservation methods with your patients who may need cyclophosphamide or who may need to postpone their pregnancy plans?	Extremely confident	19 (16%)
	Moderately confident	26 (21%)
	Slightly confident	21 (17%)
	Somewhat confident	33 (27%)
	Not at all confident	23 (19%)

Are you confident in discussing assisted reproductive techniques with your patients?	Extremely confident	18 (15)
	Moderately confident	24 (20%)
	Slightly confident	19 (16%)
	Somewhat confident	37 (30%)
	Not at all confident	24 (20%)

### Practices

The majority of the participants (93/122,77%,) reported frequently seeing patients who were told that they could never conceive as long as they were using Disease Modifying Anti Rheumatic Drugs (DMARD) and immunosuppressants. Seventy-two (59%) participants reported frequently seeing patients who have been advised against a normal vaginal delivery due to their underlying rheumatological condition. The majority of the respondents reported having faced conflicts with other specialties regarding drug safety in pregnancy. Most (95%) believed frequent communication between the obstetrician and rheumatologist is important for successful management (**Table 4**).

**Table 4.** Responses to questions in the Practices domain.

**Characteristics**	**Answers**	**N (%)**

Typically, when do you initiate the topic of pregnancy plans with your patient?	Every patient of childbearing age	94 (77%)
	Only if initiating methotrexate, cyclophosphamide or leflunomide	24 (20%)
	Discuss if initiated by the patient	02 (2%)
	If they plan marriage/family	01 (1%)
	Whenever indicated	01 (1%)

Have you encountered patients who have been told they can never conceive as long as they are using DMARDs and immunosuppressants (of any kind, for any condition, irrespective of the disease being in remission?)	Often	47 (39%)
	Sometimes	46 (38%)
	Rarely	19 (16%)
	Never	10 (8%)

Have you encountered patients who have been told that they can never have a normal delivery due to their rheumatological condition and will need a Caesarean section at the earliest (typically 37 weeks?)	Often	24 (20%)
	Sometimes	48 (39%)
	Rarely	31 (25%)
	Never	19 (16%)

Have you faced conflicts with other specialties regarding doses and drugs in patient planning or currently pregnant?	Often	43 (35%)
	Sometimes	55 (45%)
	Rarely	21 (17%)
	Never	03 (3%)

How frequently do you see the conferences or CMEs conducted by obstetricians/gynaecologists discussing the management of pregnancy in rheumatological conditions?	Often	02 (2%)
	Sometimes	22 (18%)
	Rarely	71 (58%)
	Never	27 (22%)

Do you agree that successful management of pregnancy in patients with rheumatologic conditions needs frequent communication between the rheumatologist and obstetrician?	Strongly Agree	91 (75%)
	Agree	25 (20%)
	Strongly disagree	06 (5%)

### Patient beliefs

A majority (84%) of rheumatologists reported that their patients had concerns about the effect of drugs on pregnancy. It was common (51%) to see patients stop all their drugs once pregnancy was confirmed. Most of them (57%) frequently came across patients who were concerned about the effect of disease on their pregnancy (**Table 5**).

**Table 5.** Responses to questions regarding patient beliefs.

**Characteristics**	**Answers**	**N (%)**

Have you encountered patients who would like to plan a pregnancy but are concerned about the effect of pregnancy on their disease?	Often	70 (57%)
	Sometimes	43 (35%)
	Rarely	08 (7%)
	Never	01 (1%)

Have you encountered patients who would like to plan a pregnancy but are concerned about the effect of drugs during pregnancy?	Often	102 (84%)
	Sometimes	18 (15%)
	Rarely	02 (2%)

How frequently have you had patients with rheumatological conditions stop all their drugs after conceiving (planned or unplanned pregnancy?)	Often	62 (51%)
	Sometimes	40 (33%)
	Rarely	17 (14%)
	Never	03 (2%)

### Rheumatologists’ opinions

Several rheumatologists (62.3%) believed that a lack of proper follow-ups during pregnancy with a rheumatologist was one of the main reasons for poor pregnancy outcomes. Most (93%) agreed that a joint fertility clinic is necessary for adequately addressing reproductive issues in rheumatology. Most (92%) respondents also felt the need for guidelines specific to the Indian population was very important (**Table 6**).

**Table 6.** Responses to questions regarding Physicians’ opinions.

**Characteristics**	**Answers**	**N (%)**

What do you think is the main contributing factor to a bad obstetric outcome in a patient with a rheumatological condition?	Lack of regular follow-ups, with the rheumatologist to detect early flares	76 (63%)
	Poor drug compliance by the patient	23 (19%)
	Improper prescription of drugs during antenatal/pregnancy	12 (10%)
	Unexpected pregnancy morbidity despite following the recommended precautions	10 (8%)

Do you agree that there is a need for a fertility clinic to discuss all reproductive issues with the patient in a multidisciplinary setting (rheumatologist and gynaecologist/obstetrician see the patient together)?	Strongly Agree	66 (54%)
	Agree	47 (39%)
	Neither agree nor disagree	06 (5%)
	Strongly disagree	03 (2%)

How important would you rate the need for Indian guidelines specifically for managing reproductive health in rheumatological patients?	Essential	54 (44%)
	Very important	58 (48%)
	Of average importance	09 (7%)
	Of little importance	01 (1%)

What factors do you think make the Indian scenario different, requiring specific guidelines?	Poor follow up	97 (80%)
	Unwilling for routine checks and investigations	97 (80%)
	Lack of Literacy	86 (71%)
	Poor understanding of the diseases	86 (71%)
	Lack of adequate health insurance and cost burden	80 (66%)
	Less number of rheumatologists in the vicinity	78 (64%)
	The social stigma of having a chronic health condition	73 (60%)
	Mistrust of allopathic doctor	57 (47%)

What effective patient information methods would you recommend to improve awareness regarding pregnancy and rheumatological conditions?	One-on-one counselling at the OP visit	72 (60%)
	Family counselling	25 (21%)
	Patient support groups	08 (15%)
	Pamphlets distribution	05 (4%)
	Website and social media	26 (25%)

Would you like to suggest any other methods to improve reproductive health in rheumatology?	Increase awareness among obstetricians, doctors	23 (19%)
	Regular CMEs	07 (6%)
	Teamwork (Rheumatologist and obstetrician)	14 (11%)

## DISCUSSION

This survey was carried out to assess the knowledge, awareness, and practices relevant to reproductive rheumatology among Indian rheumatologists. This survey reveals several areas with lacunae and scope for improvement as well as many interesting points with potential global relevance.

In the present survey, 24% of the respondents reported being less or not at all familiar with the ACR 2020 and BSR 2023 guidelines for management in pregnancy and lactation. A Canadian survey among 98 rheumatology trainees and 44 programme directors to assess interest and knowledge in reproductive health revealed high interest but less confidence in this topic. A substantial proportion (38% of trainees and 24% of programme directors) were unaware of the ACR 2020 guidelines regarding reproductive health.^6^ Approximately 15% of respondents (n=18) in our survey were not accurate in selecting appropriate contraceptive choices.

In another interview-based study on 12 subjects, rheumatologists expressed discomfort in discussing family planning and contraception with patients and expected the gynaecologists to prescribe contraceptive advice.^4^ A survey by Clowse et al. on rheumatologists’ knowledge about contraception, teratogens, and pregnancy risks noted that though most were skilled in managing lupus pregnancies, there were gaps in knowledge regarding contraception and drug safety.^3^ These studies demonstrate the growing recognition of reproductive health in the comprehensive care of the patient, however, lacunae in the delivery of the care pathway need to be addressed.

In our study, the participants were asked to classify Prednisolone as A, B, C, D, or X based on the 2015 USFDA Pregnancy Risk Categories. Only 38% of respondents answered this question correctly. Regarding drug compatibility with lactation, scope for improvement was evident as 18% were under the impression that azathioprine, colchicine, and tacrolimus were incompatible with lactation. A substantial proportion (36%) reported either no or only slight familiarity with the more recent PLLR classification of drugs. A study by Mills et al showed that there were knowledge gaps about lactation-compatible and incompatible medication among rheumatologists (78% and 65% respectively) and non-rheumatology providers (31% and 46% respectively). When they were re-surveyed 5 months after providing them with a pocket-sized lactation medication information card, there was a significant increase in the knowledge (over 90%) in both groups.^7^ This underscores a potential for improvement in rheumatologists’ understanding of drug safety. Simple measures such as readily accessible information cards, summary sheets, question banks, didactics, and online modules may serve this purpose.^6^

Our survey showed that rheumatologists were less confident in discussing ART and oocyte preservation techniques. This supports the need for education regarding guiding patients for ART, prerequisite screening, timing, and drug modification.^8^ A survey of rheumatologists and dermatologists managing psoriatic arthritis in three central European countries revealed that the former engage with many more specialties and are generally more aware of treat-to-target strategies, drug safety, and vaccination recommendations relating to the newborns of their pregnant and postpartum patients receiving biologics.^9^ An Australian study showed that gastroenterologists were better than obstetricians and primary care physicians regarding the knowledge of inflammatory bowel disease (IBD) pregnancy-related topics, including medication.^10^ Although patients may approach their obstetricians for advice on the continuation of drugs during pregnancy, rheumatologists may be better equipped to manage biologics and DMARD use in pregnancy and lactation, close collaboration, therefore is essential.

In our survey, rheumatologists reported frequently having come across patients who have received varying information such as the inability to conceive due to the diagnosis of the rheumatological condition, while on any DMARDs, and the impossibility of having a normal vaginal delivery. It has been reported that nearly one-third of patients remain childless after a diagnosis of rheumatological conditions.^11^ According to a survey conducted by The Pregnancy and Lactation Autoimmune Network (PLAN) Registry on 154 patients with AIRDs, half of women with Systemic Lupus Erythematosus (SLE) chose not to have children, with the most commonly expressed reason being concern over the potential adverse effects of DMARD therapy on their unborn babies.^12^ Nearly half of women with different AIRD were worried that if they became pregnant or breastfed, their disease would be passed on to them.^13^ Most guidelines recommend a remission period of 6 months before planning a pregnancy.^1,2^ The delayed pregnancies compounded with patients’ fears and misinformation about pregnancy may increase levels of voluntary childlessness and smaller family sizes.

In our study also rheumatologists reported that they frequently faced conflicts with other specialists while managing these patients. Patients’ nonadherence to drugs and medical advice, ambiguity or inability to plan pregnancies make drug modifications challenging for rheumatologists, especially in the setting of active disease. Patients are in general mistrustful of drug usage during pregnancy and may rely more on their obstetrician or primary care physician for advice on drug continuation. While access to healthcare is usually not a problem, there is a vast variability in the services provided by the healthcare system. Lack of infrastructure, inconsistencies in guidelines in various specialties, and lack of team support are healthcare system-related issues contributing to clinical inertia.^14,15^ One of the studies on DMARD use in pregnancy by obstetricians and rheumatologists showed disparities in practices by specialists regarding the continuation or stoppage of drugs.^16^ Another web-based survey conducted on 121 physicians found heterogeneity in the postpartum management of patients, in the use of low-dose aspirin and contraception advice.^17^ This underscores the need for close collaboration between various specialties, common guidelines, and physician education so that the patient can receive consistent and standardised information.

We also explored challenges specific to the Indian scenario with potential global relevance. Poor outcomes, according to the majority of rheumatologists (59%), were primarily caused by irregular follow-ups with them. Twenty -nine of the respondents reported poor adherence to medications as another cause, while half stated that patients frequently stopped taking their medications soon after pregnancy was confirmed without seeing their rheumatologist. These results, in general, were consistent with previous literature.^18^ Drug compliance may be poor during pregnancy due to anxiety regarding the adverse effects of medication on the foetus, misinformation from varied sources, or symptomatic improvement leading to early stoppage of drugs. However, it is important to acknowledge that the patient’s desire for pregnancy may override their aspirations for health.

While enquiring into preferred methods of patient education on issues related to the management of pregnancy, it is worth noting that the majority of the respondents opted for a direct discussion with the patient (70/122, 57%) as opposed to counselling for the entire family, patient support groups, or pamphlet distribution. In India, women may not always be able to make autonomous decisions in reproductive issues due to various societal barriers and familial hierarchy. Legalities regarding the decision to not have children, if not accepted by both spouses, are vague at best and may even be considered grounds for mental cruelty. These aspects underscore a need for the dissemination of information on reproductive health to the general public, healthcare professionals, and policy makers.^19^

The patients’ and/or their family’s decisions regarding reproductive health may not always be aligned with the rheumatologists. It may translate into poor communication and decision-making. Frank and factual communication taking into consideration the patient’s viewpoint, including cultural beliefs into account and consistent information imparted across various specialties are essential for better patient compliance and decision-making.^20^ Most of the respondents (113/122, 93%) in our survey agreed that a multidisciplinary approach with the obstetrician is useful in managing patients. As most of our respondents (n=73, 60%) had obstetric services available full-time, a blueprint to begin combined clinics appears to be the need of the hour.

To the best of our knowledge, this is the first study evaluating the rheumatologists’ perspective on reproductive health from India. A study conducted in the Asia Pacific region looking at clinicians’ and patients’ perspectives found similar lacunae in the clinicians’ knowledge and variations in targets of clinical practice.^21^ Misconceptions about drug safety were common in patients and a need for both clinicians’ and patients’ education was demonstrated.^21^

Limitations of the study include the small number of respondents and bias related to self-reporting. As the questionnaire was disseminated through various social media and group emails to various rheumatologists, and participation was voluntary, we could not calculate the response rates to the survey. A repeat questionnaire conducted after an educational conference or meeting would help in improving knowledge and confidence. Our survey was aimed at rheumatologists, a survey directed to both obstetricians and rheumatologists would better inform the views of each specialty. Similarly, another survey of the patients would help in understanding their viewpoint better. There may have been a possible selection bias as only those interested in or dealing with reproductive rheumatology may have chosen to participate. This may have biased the results too, leading to more correct responses.

Developing nations have different challenges in the practices of rheumatology, including infrastructure, availability of specialists, availability of diagnostics, access to therapies, patient education, and research challenges.^22^ Until such area-specific data is available, formulating guidelines that are adapted to the region will be challenging. Our study contributes to the sparse literature available from Southeast Asia in reproductive rheumatology.

In conclusion, the results of our survey reflect the practical experience of rheumatologists. It underscores a scope for improvement at various levels for patients, the general public, clinicians, and the healthcare system. A multidisciplinary approach with well-defined roles for each specialty and collaboration between them combined with relevant patient education are essential for effective care. Improving the healthcare infrastructure and training of personnel can help mitigate time constraints. Future work can focus on scrutinising patients’ perspectives, which shall help elucidate their specific priorities, concerns, and counselling preferences.

## CONFLICT OF INTEREST

The authors declare no conflict of interest.

## APPENDIX

### Knowledge, attitude, and practices of Rheumatologists in the field of reproductive rheumatology

Managing pregnancy in persons with rheumatological conditions is especially challenging. The purpose of this survey is to assess the baseline knowledge and attitudes of rheumatologists caring for reproductive issues in their patients. Your email address, phone number, and other details regarding your practice will remain confidential.

Baseline Demographics:

1. What is your gender?
  - Male
  - Female
  - Prefer not to say
2. What is your age (in years)
  - 31–40
  - 41–50
  - 51–60
  - >60
  - Others:
3. Please enter your city of practice?
4. What is your predominant area of practice?
  - Urban
  - Rural
5. Which amongst the following best describes your job?
  - Rheumatologist mainly seeing adult patients
  - Paediatric Rheumatologist
  - DM/DNB resident undergoing training in rheumatology
  - Other:
6. How many years of experience do you have?
  - < 3
  - 3–7
  - >7
7. What is the setup you primarily practice in?
  - Corporate hospital
  - Teaching institute
  - Private clinic
  - Other:
8. Are you actively involved in academic and research activities?
  - Yes
  - No
9. Does your hospital or clinic also have an Obstetrician working full time?
  - Yes
  - No
10. On average, how many patients with rheumatic diseases and reproductive issues do you see per month?
  - <3
  - 3–7
  - >7
11. What is the most common rheumatic disease that you encounter in pregnant patients?
  - Rheumatoid Arthritis
  - Systemic Lupus Erythematosus
  - Antiphospholipid Antibody Syndrome (APLA)
  - Spondyloarthritis
  - Other:
  Knowledge about the Topic
12. Are you familiar with ACR 2020 guidelines for the management of reproductive health in rheumatic and musculoskeletal diseases?
  - Not at all familiar
  - Slightly familiar
  - Somewhat familiar
  - Moderately familiar
  - Extremely familiar
13. Are you familiar with the Pregnancy and Lactation Labelling Final Rule (PLLR) as defined by US FDA on the prescription of drugs and biologics during pregnancy and breastfeeding?
  - Not at all familiar
  - Slightly familiar
  - Somewhat familiar
  - Moderately familiar
  - Extremely familiar
14. Are you familiar with the BSR 2022 guidelines on prescribing drugs in pregnancy and breastfeeding?
  - Not at all familiar
  - Slightly familiar
  - Somewhat familiar
  - Moderately familiar
  - Extremely familiar
15. Which USFDA category for the safety of drugs in pregnancy does Prednisolone come under?
  - Category A
  - Category B
  - Category C
  - Category D
  - Category X
16. Which of the following methods of Contraception is preferred in APLA patients?
  - Combined Estrogen and Progesterone Pill
  - Progesterone Intra Uterine Device
  - Depot Medroxy Progesterone Acetate
  - Transdermal Patch
  - Other:
17. Which of the following drugs is not compatible with breastfeeding?
  - Mycophenolate Mofetil
  - Azathioprine
  - Tacrolimus
18. For which among the following patients do you routinely screen for anti-R0 and anti-La antibodies before planning pregnancy?
  - All Patients with arthritis and connective tissue diseases
  - Sjogren's syndrome
  - Other:
  Confidence and attitude of the Rheumatologist
19. How confident are you in managing pregnancy in rheumatological conditions
  - Not at all familiar
  - Slightly familiar
  - Somewhat familiar
  - Moderately familiar
  - Extremely familiar
20. How confident are you in using Biologics during pregnancy?
  - Not at all familiar
  - Slightly familiar
  - Somewhat familiar
  - Moderately familiar
  - Extremely familiar
21. Are you confident in discussing contraception methods with your patients?
  - Not at all familiar
  - Slightly familiar
  - Somewhat familiar
  - Moderately familiar
  - Extremely familiar
22. Are you confident in discussing oocyte preservation methods with your patients who may need cyclophosphamide or who may need to postpone their pregnancy plans?
  - Not at all familiar
  - Slightly familiar
  - Somewhat familiar
  - Moderately familiar
  - Extremely familiar
23. Are you confident in discussing artificial reproductive techniques with your patients?
  - Not at all familiar
  - Slightly familiar
  - Somewhat familiar
  - Moderately familiar
  - Extremely familiar
  Practices of the Rheumatologist
24. Typically, when do you initiate the topic of pregnancy plans with your patient?
  - Every patient, of the childbearing age
  - Only if initiating methotrexate or cyclophosphamide or leflunomide
  - Discuss if initiated by the patient
  - Other:
25. Have you encountered patients who have been told they can never conceive as long as they are using DMARDs and immunosuppressants (of any kind, for any condition, irrespective of the disease being in remission?)
  - Never
  - Rarely
  - Sometimes
  - Often
26. Have you encountered patients who have been told that they can never have a normal delivery due to their rheumatological condition and will need a Caesarean section at the earliest (typically 37 weeks?)
  - Never
  - Rarely
  - Sometimes
  - Often
27. Have you faced conflicts with other specialties regarding doses and drugs in patient planning or currently pregnant?
  - Never
  - Rarely
  - Sometimes
  - Often
28. How frequently do you see the conferences or CMEs conducted by obstetricians/gynaecologists discussing the management of pregnancy in rheumatological conditions?
  - Never
  - Rarely
  - Sometimes
  - Often
29. Do you agree that successful management of the pregnancy in patients with rheumatologic conditions needs frequent communication between the rheumatologist and obstetrician?
  - Strongly disagree
  - Disagree
  - Neither agree or disagree
  - Agree
  - Strongly agree
  Patient beliefs
30. Have you encountered patients who would like to plan a pregnancy but are concerned about the effect of pregnancy on their disease?
  - Neve
  - Rarely
  - Sometimes
  - Often
31. Have you encountered patients who would like to plan a pregnancy but are concerned about the effect of drugs during pregnancy?
  - Never
  - Rarely
  - Sometimes
  - Often
32. How frequently have you had patients with rheumatological conditions stop all their drugs after conceiving (planned or unplanned pregnancy?)
  - Never
  - Rarely
  - Sometimes
  - Often
  Physician opinions
33. What do you think is the main contributing factor in a bad obstetric outcome in a patient with a rheumatological condition - please select one option.
  - Improper prescription of drugs during antenatal/pregnancy
  - Poor drug compliance by the patient
  - Lack of regular follow-ups, with a rheumatologist to detect early flares
  - Unexpected pregnancy morbidity despite following the recommended precautions
34. Do you agree that there is a need for a fertility clinic to discuss all reproductive issues with the patient in a multidisciplinary setting (rheumatologist and gynaecologist/obstetrician see the patient together)?
  - Strongly disagree
  - Disagree
  - Neither agree or disagree
  - Agree
  - Strongly agree
35. How important would you rate the need for Indian guidelines specifically for managing reproductive health in rheumatological patients?
  - Not Important at all
  - Of Little Importance
  - Of Average Importance
  - Very Important
  - Essential
36. What factors do you think make the Indian scenario different, requiring specific guidelines - you can select multiple options
  Check all that apply.
  - Mistrust of allopathic doctors
  - Poor follow-up, unwilling for routine checks and investigations
  - Poor understanding of the diseases, due to literacy
  - Lack of adequate health insurance and cost burden
  - Social stigma on having a chronic health condition
  - Family/societal pressure not to take too many medicines as they are harmful
  - Less number of rheumatologists in the vicinity, as they are mostly concentrated in the urban cities
37. Would you be willing to contribute data regarding pregnancies in your rheumatological patients to an online database?
  - No
  - Maybe
  - Yes
38. What effective patient information methods would you recommend improving awareness regarding pregnancy and rheumatological conditions?
  - One-on-one counselling at the OP visit, as it may be a sensitive issue
  - Family Counselling
  - Pamphlets distribution
  - Refer to specific websites for further information
  - Patient support groups
  - Other:
39. Would you like to suggest any other methods to improve reproductive health in rheumatology?
